# Exposure to household air pollution from solid cookfuels and childhood stunting: a population-based, cross-sectional study of half a million children in low- and middle-income countries

**DOI:** 10.1093/inthealth/ihab090

**Published:** 2022-01-12

**Authors:** Rishi Caleyachetty, Nakawala Lufumpa, Niraj Kumar, Nuredin Ibrahim Mohammed, Hana Bekele, Om Kurmi, Jonathan Wells, Semira Manaseki-Holland

**Affiliations:** Nuffi eld Department of Medicine, University of Oxford, Oxford, OX3 7BN, UK; Warwick Medical School, University of Warwick, Coventry, CV4 7HL, UK; Institute of Applied Health Research, University of Birmingham, Birmingham, B15 2TT, UK; University College London Medical School, University College London, London, WC1E 6DE, UK; Medical Research Council Unit The Gambia at the London School of Hygiene & Tropical Medicine, Atlantic Boulevard, Fajara, P.O. Box 273, Banjul, The Gambia; World Health Organization, Inter-Country Support Team, Zimbabwe WHO Country Office, Harare, Zimbabwe; Faculty of Health and Life Sciences, Coventry University, Coventry, CV1 5FB, UK; Department of Medicine, McMaster University, Hamilton, ON, L8S 4L8, Canada; Childhood Nutrition Research Centre, Population Policy and Practice Research and Teaching Department, UCL Great Ormond Street Institute of Child Health, London, WC1N 1EH, UK; Institute of Applied Health Research, University of Birmingham, Birmingham, B15 2TT, UK

**Keywords:** child growth, demographic and health surveys, indoor air pollution, stunting

## Abstract

**Background:**

Household air pollution from the incomplete combustion of solid cookfuels in low- and middle-income countries (LMICs) has been largely ignored as a potentially important correlate of stunting. Our objective was to examine the association between solid cookfuel use and stunting in children aged <5 y.

**Methods:**

We used data from 59 LMICs’ population-based cross-sectional demographic and health surveys; 557 098 children aged <5 y were included in our analytical sample. Multilevel logistic regression was used to examine the association between exposure to solid cookfuel use and childhood stunting, adjusting for child sex, age, maternal education and number of children living in the household. We explored the association across key subgroups.

**Results:**

Solid cookfuel use was associated with child stunting (adjusted OR 1.58, 95% CI 1.55 to 1.61). Children living in households using solid cookfuels were more likely to be stunted if they lived in rural areas, the poorest households, had a mother who smoked tobacco or were from the Americas.

**Conclusions:**

Focused strategies to reduce solid cookfuel exposure might contribute to reductions in childhood stunting in LMICs. Trial evidence to assess the effect of reducing solid cookfuel exposure on childhood stunting is urgently needed.

## Introduction

While significant progress has been made towards reducing the global prevalence of childhood stunting, the prevalence of childhood stunting in many countries remains unacceptably high with negative consequences for those children affected.^1^ Child stunting has decreased at approximately 1.8% per year globally and over the next decade will reflect a decrease by only 18%.^2^ Consequently, the goal set by the World Health Assembly (WHA) will not be achieved. Further reductions in child stunting will require a broader strategy that incorporates a wider set of risk factors.^2^

Approximately 2.8 billion people, mostly in low- and middle-income countries (LMICs), are exposed to household air pollution from the incomplete combustion of solid fuels traditionally used for cooking (e.g. wood, agricultural residue, dung, charcoal and coal).^3^ Household members, particularly young children, are exposed to pollutants, including particulate matter, carbon monoxide, black carbon and polycyclic aromatic hydrocarbons.^4^ Children aged <5 y are uniquely vulnerable to exposure to household air pollution for several reasons. They stay indoors, spending a large proportion of the time in the kitchen,^5^ and are often carried on their mother's back or lap while cooking.^6^ Young children also inhale more air than adults each day on a per kilogram body-weight basis.^7^

Household air pollution has recently been suggested to affect child growth.^8^ This may be through several mechanisms that reflect either a direct effect of airborne particulate exposure on growth or indirectly through increased morbidity.^9–11^ Exposure to pollutants is associated with disruptions to the endocrine system, which regulates growth.^11–13^ Additionally, air pollution exposure increases the risk of acute respiratory infections, which disrupt growth through increased metabolic demand.^14,15^

A 2018 systematic review identified seven studies examining the association between child stunting and household air pollution from solid cookfuels in LMICs, using cross-sectional and cohort data.^16^ Existing evidence about the association between child stunting and exposure to household air pollution is mixed. Previous studies were limited by small sample sizes, varying measures of exposure to household air pollution, improper model adjustments for confounders and limited subgroup analysis. Using nationally representative and comparable demographic and health surveys (DHS) in 557 098 children aged <5 y from 59 LMICs, we examined the association between solid cookfuel use and childhood stunting.

## Materials and Methods

### Data source

We used DHS, which are nationally representative cross-sectional household surveys conducted at approximately 5-y intervals in LMICs. We assessed the most recent DHS data, from January 2000 to date, with available data on the use of cookfuel and height-for-age. Standardised methodology and measurement tools have been developed for the collection of DHS data in each country.

The DHS use a stratified two-stage random sampling approach. Census enumeration areas are identified based on a probability proportional to the sampling area. Within each of the selected enumeration areas, a random selection of households is identified from a complete listing of households. In each sampled household, all consenting women aged 15–49 y are interviewed, and their children aged 0–60 mo undergo anthropometric measurements including height, from which stunting can be assessed. Our analytical sample included alive children aged <5 y with valid height measurements and living with their mother who is a de jure resident.

All the women included in the DHS provided written consent for themselves and their children. The DHS received ethical approval centrally by ICF International (Calverton, MD, USA) institutional review board and locally by individual review boards within every participating country.

### Stunting

DHS include data about each child's age (in months and years) and measured length/height. We measured stunting according to the WHO reference anthropometric measurements for children.^17^ Height-for-age z-scores (HAZ) were used to measure whether a child was stunted or not. HAZ indicate the number of standard deviations a child's height is from the median height-for-age in the reference population. A child with a z-score <–2 was categorised as stunted.

### Solid cookfuel use

Each respondent was asked ‘What type of fuel does your household mainly use for cooking?’ Responses included electricity, electricity from other source, liquefied petroleum gas (lpg), natural gas, biogas, kerosene, coal lignite, charcoal, wood, straw shrubs, agriculture crop, animal dung, cardboard/paper and solar power. Solid cookfuel use was defined as using the following fuels: coal lignite, charcoal, wood, straw shrubs, agriculture crop, animal dung and cardboard/paper. Additionally, we constructed a three-category variable based on the cleanliness of the cookfuel: clean (electricity, electricity from other source, lpg, natural gas, biogas and solar), moderately clean (kerosene) and not clean (coal lignite, charcoal, wood, straw shrubs, agriculture crop, animal dung and cardboard/paper).^18^

### Confounders

Based on a priori subject matter knowledge and the literature, we adjusted for the following confounders: child sex, child age in months, maternal education and the number of children in the household.^19,20^ Child sex was recorded as either male or female. Child age in months was calculated from the date of birth. Maternal education was self-reported and categorised in three groups: none (no formal education), primary (any primary education, including completed primary education) and secondary or higher (any secondary education, including complete secondary).

We hypothesized that the prespecified variables, including the residence type, location of kitchen, household wealth, exclusive breastfeeding for 6 mo, maternal tobacco smoking, birth weight and WHO region, may alter the magnitude of the association between solid cookfuel use and child stunting. Urban or rural residence was categorised according to country-specific delimitations at the time of the survey. The location of the kitchen was categorised as outdoors or indoors. The household wealth index was derived using principal component analyses of household assets and characteristics of the building, presence of electricity, water supply and sanitary facilities, in addition to other variables associated with wealth.^21^ The score is provided with the original survey datasets and calculated according to a standard methodology.^21^ Household wealth was categorised into quintiles (poorest, poorer, middle, richer or richest). Exclusive breastfeeding was defined for all children in the first 6 mo and was assessed from the question: ‘Are you currently breastfeeding [name of the child]?’ A ‘Yes’ response led to further questions on additional food and liquid given to the child in the past 24 h. We categorised children as exclusively breastfed if they had been breastfed in the 24 h preceding the survey and had not been fed any other type of food. Maternal smoking in the DHS is assessed via questionnaire. Participants were asked four questions, which are answered either ‘Yes’ or ‘No’ regarding current cigarette, pipe or other country-specific tobacco usage. We classified any ‘Yes’ response to the use of smoking products as ‘maternal smoking’, creating a binary variable. The DHS record birth weight in kilograms according to health card records or mother's recall. As per the WHO classification, birth weight was categorised as low birth weight (<2500 g) and normal birth weight (≥2500 g). Regions, according to WHO categorisations, are the Americas, African, European, Eastern Mediterranean, South-East Asian and Western Pacific.

### Statistical analysis

We pooled individual-level data from the DHS and created a sample grouped into a three-level hierarchical structure. Children formed level 1, nested within communities at level 2 and countries at level 3. To account for the complex survey design, we used multilevel logistic regression models to estimate the association between solid cookfuel use and childhood stunting. The association between cleanliness of cookfuels (clean, moderately clean, not clean) and childhood stunting was also examined. We present adjusted OR (AOR) and 95% CI. All our models adjusted the following a priori confounders: child age, child sex, maternal education and number of children living within the household. Random effects at level 2 and level 3 were also controlled for.

Interaction tests between solid cookfuel use and the subgroups (residence type, kitchen location, household wealth, exclusive breastfeeding, maternal smoking, birth weight and WHO regions) were performed by including a solid cookfuel use × subgroup interaction term in the model. We report p-values for tests of interaction as well as present subgroup-specific AOR estimates. All models were adjusted for child age, child sex, maternal education and number of children living within the household.

Our multilevel models did not weight the data, because DHS sample weights are country-specific and not suitable for multilevel analysis. However, we repeated analyses using a two‐stage individual participant data meta‐analysis approach,^22^ preserving country-specific sample weights and obtained similar OR estimates and 95% CIs.

Stata/SE version 16.1 (StataCorp, College Station, TX, USA) was used for data cleaning and preparation. Multilevel models were run in MLwiN 3.05 using the runmlwin program in Stata 16.1. Multilevel model parameters were estimated using iterative generalised least squares and marginal quasi-likelihood algorithms.

## Results

Datasets from 2000 to 2018 DHS were available for 69 LMICs. Of these, 59 (86%) country datasets included data on self-reported primary fuel used for cooking and HAZ, and were included in our analysis. According to WHO regions, the following number of LMICs were included in our analysis: 41 out of 45 in Africa; 3 out of 16 in Eastern Mediterranean; 3 out of 20 in Europe; 7 out of 25 in the Americas; 4 out of 11 in South-East Asia; and 1 out of 18 in Western Pacific.

**Figure 1.**
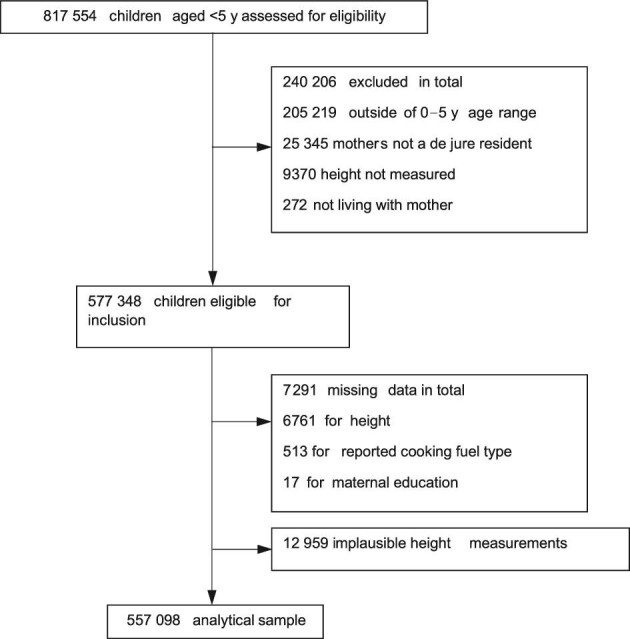
Sample selection.

In total, 577 348 children were eligible for inclusion in our analysis (Figure 1). Of these children, 20 233 (3.5%) were excluded due to missing (n=12 959) or implausible (n=6761) data on height-for-age, and missing data on reported cookfuel type (n=513). A further 17 children were excluded due to missing values for maternal education. The analytical sample was based on the remaining 557 098 children (96% of the total eligible population).

**Figure 2.**
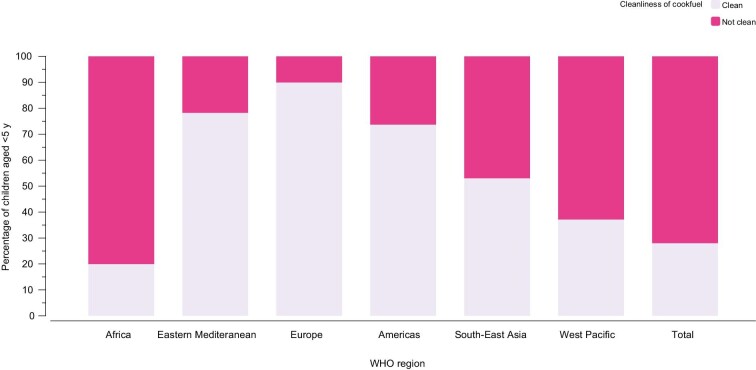
Proportion of children living in households using clean cookfuels by WHO region.

The mean child age was 29 (range 0–60) mo and 51.2% (n=285 188) were boys (Table 1). A total of 34% of children were stunted (ranging from 7.9% in the Dominican Republic to 54.6% in Burundi). The most common cookfuel used in households was wood (55.0%) (Supplementary Table 1). The majority of children (72.0%) lived in households using solid cookfuels (Figure 2), ranging from 0.0% in Jordan to 99.9% in Sierra Leone (Supplementary Tables 1 and 2). Regionally, the proportion of children living in households using solid cookfuels was highest in Africa (46.8%) and lowest in Europe (0.9%) (Figure 2).

**Table 1.** Characteristics of DHS

Country	Year	Children aged <5 y (n)	Analytical sample (n)	Mean age (mo)	Living in urban area (%)	Lowest household wealth quintile (%)	No maternal education (%)	Stunting (%)
Albania	2017–2018	2762	2459	26.2	40.6	32.9	0.9	12.9
Angola	2015–2016	14 322	6296	27.1	54.8	23.8	34.8	37.5
Armenia	2015–2016	1724	1573	27.0	55.6	20.5	5.4	10.6
Azerbaijan	2006	2297	1957	27.1	46.8	25.9	1.2	27.6
Bangladesh	2014	7886	6416	27.3	31.8	22.3	16.1	37
Benin	2017–2018	13 589	11 477	27.7	40.7	21.3	65.1	31.7
Bolivia	2008	8605	7685	27.7	51.4	27.9	5.5	26.5
Burkina Faso	2010	15 044	6582	27.6	21.9	20.1	82.8	34.4
Burundi	2016–2017	13 192	6021	27.8	15.6	20.2	45.7	54.6
Cambodia	2014	7165	4331	28.8	27.2	24.2	14.1	32.7
Cameroon	2018	9733	4254	28.2	45.4	19.9	23.7	28.4
Chad	2014	18 623	9893	29.9	20.7	19.5	72	42.8
Colombia	2010	17 756	15 935	28.2	62.9	37.4	3	14.5
Comoros	2012	3149	2526	28.8	33.4	27.3	46.5	28.9
Congo	2011–2012	9329	4272	28.1	25.3	45	9.6	26.9
Cote d'Ivoire	2011–2012	7776	3151	28.1	33.5	26.3	67.5	30.1
Dominican Republic	2013	3714	3067	28.3	70.1	29.5	3.4	7.9
DR Congo	2013–2014	18 716	8080	28.0	28.9	27.4	21.7	44.2
Eswatini	2006	2812	2010	29.9	21	22.3	9.5	27.1
Ethiopia	2016	10 641	8767	28.4	18.3	36	64	36.3
Gabon	2012	6067	3343	28.4	60.8	46.7	6.6	23.9
Gambia	2013	8088	3155	28.4	31.4	25.9	64.2	25.9
Ghana	2014	5884	2669	28.4	40.4	33	36.2	19.2
Guatemala	2014–2015	12 440	11 603	28.5	34.2	27.2	18.3	46.6
Guinea	2018	7951	3405	28.4	28.3	25	77.4	31.1
Guyana	2009	2178	1616	28.5	19.1	41.8	3.4	23.8
Haiti	2016–2017	6530	5531	28.6	28.4	30.9	20.8	21.5
Honduras	2011–2012	10 888	9656	28.5	32.9	33.8	5.9	25.6
India	2015–2016	259 627	219 908	28.7	23.8	26.2	31.2	38.1
Jordan	2012	10 360	6074	28.7	68.9	26.9	2.8	8.9
Kenya	2014	20 964	18 403	28.8	31.5	34.8	22	27.3
Kyrgyz Republic	2012	4363	3869	28.8	25.1	22	0	18.4
Lesotho	2014	3138	1248	28.9	22.6	27.2	1.4	35
Liberia	2031	7606	3163	28.9	31.6	35.7	47.9	31.5
Madagascar	2008–2009	12 448	5198	29.1	17.9	28.9	28.3	48
Malawi	2015–2016	17 286	5116	29.2	16.1	21.8	12.5	35.3
Maldives	2016–2017	3106	2344	29.1	7.7	28.8	1.5	15.1
Mali	2018	9940	8234	29.1	24.4	19.6	72.5	26.7
Moldova	2005	1552	1295	29.1	51	16.5	0.6	10.5
Morocco	2003–2004	6180	5421	28.9	43.1	27.5	65.5	23.8
Mozambique	2011	11 102	9334	29.4	31.4	18.5	35	39.8
Namibia	2013	5046	1787	29.5	40.7	24.2	8.8	22.4
Nepal	2016	5038	2180	29.5	56.4	25.4	34	36.4
Nicaragua	2001	6986	5939	29.4	43.6	N/A	24.9	27.1
Niger	2012	12 558	4896	29.4	21.7	18.2	83.3	41.8
Nigeria	2018	33 924	11 160	29.4	39	19.9	38.3	36.3
Pakistan	2017–2018	12 708	3997	29.6	45.3	21.2	52	38
Peru	2012	9620	8897	29.6	57.9	29	3.7	20.7
Rwanda	2014–2015	7856	3532	29.7	21.7	24.5	14	37.8
Sao Tome and Principe	2008–2009	1931	1585	29.9	38.6	24.2	5.7	28.8
Senegal	2018	18 904	5856	29.8	28.9	33.3	67.2	20.7
Sierra Leone	2013	11 938	4300	29.8	28.8	23.3	69.3	37.8
South Africa	2016	3548	1079	30.5	46.8	26	2	26
Tajikistan	2017	6195	5707	30.0	32.9	17.9	2.4	18.5
Tanzania	2015–2016	10 233	8619	30.1	22.6	23.5	22.1	33.8
Timor-Leste	2016	7221	5851	30.0	29.4	20.2	24.8	45.7
Togo	2013	6979	3143	30.0	27	31.8	46	28.4
Uganda	2016	15 522	4308	30.3	16.8	27.1	13.1	28.4
Yemen	2013	16 093	13 580	30.3	22.8	22.3	56.2	46.2
Zambia	2018	9959	8572	30.6	29.4	28.5	10.6	35
Zimbabwe	2015	6132	4773	31.5	35.9	21.9	1.1	25.9

**Figure 3.**
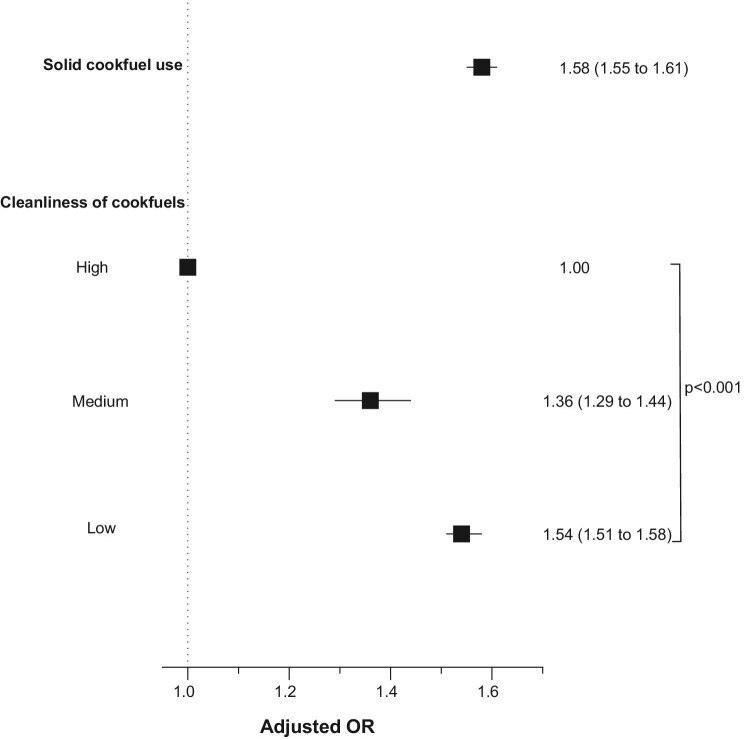
Association of solid cookfuel use and cleanliness of cookfuels with childhood stunting. Models were adjusted for child age, child sex, maternal education and number of children living within the household.

Children living in households using solid cookfuels were more likely to be stunted (AOR 1.58, 95% CI 1.55 to 1.61; p<0.0001) than children living in households not using solid cookfuels (Figure 3). Less clean cookfuels were associated with increasing odds of childhood stunting in a monotonic and linear manner (p_trend_<0.0001) (Figure 3).

**Figure 4.**
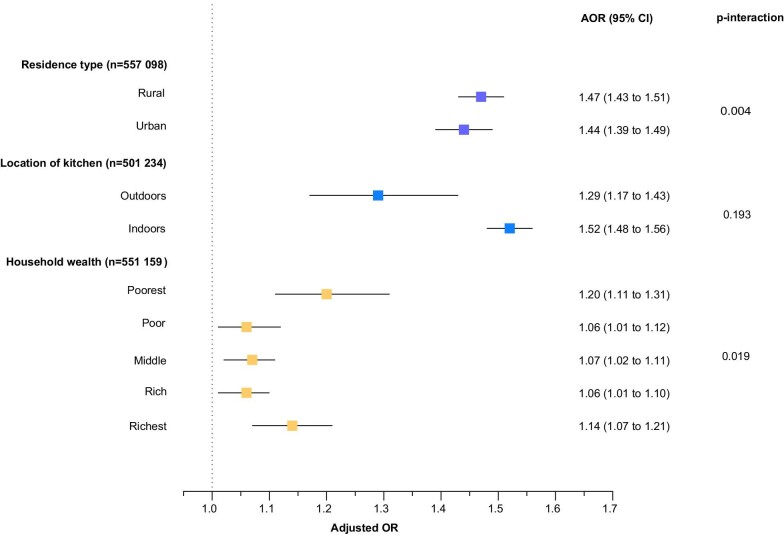
Association of solid cookfuel use with child stunting by residence type, location of kitchen and household wealth. Models were adjusted for child age, child sex, maternal education and number of children living within the household.

**Figure 5.**
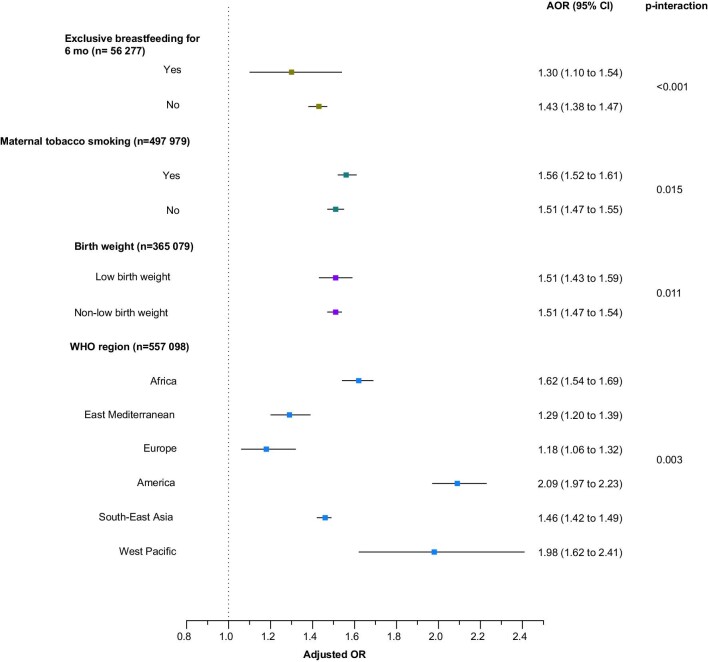
Association of solid cookfuel use with child stunting by breastfeeding status, maternal tobacco smoking status, birth weight and region. Models were adjusted for child age, child sex, maternal education and number of children living within the household.

Analyses were repeated for subgroups according to urban/rural residence, location of kitchen, household wealth, exclusive breastfeeding, maternal smoking, birth weight and WHO regions (Figures 4 and 5). Children living in households using solid cookfuels were more likely to be stunted if they were living in rural areas (p-interaction=0.004), lived in the poorest household (p-interaction=0.019), belonged to a mother who smoked tobacco (p-interaction=0.0001), were low birthweight babies (p-interaction=0.010) and living in the Americas (p-interaction=0.003).

## Discussion

To the best of our knowledge, this is the largest analysis of solid cookfuel use and child stunting to date, covering >500 000 children in 59 LMICs. Our findings demonstrate that children who lived in households primarily using solid cookfuels were more likely to be stunted, with an increased likelihood of stunting with increasing exposure to solid cookfuels.

The evidence base on the association between solid cookfuel use and stunting in children aged 0–5 y has been limited and inconsistent.^16,23^ Bruce et al.’s^23^ systematic review and meta-analysis indicated that children exposed to household air pollution from solid cookfuels were more likely to be stunted. However, this was based on only three studies: two were based on data from India and the other using DHS data for seven countries. Vilcins et al.’s^16^ systematic review identified the same three studies, with an additional study identified that was conducted in Swaziland.^24^ This analysis reported no association between exposure to solid cookfuel use and child stunting. Using cohort data from the first (2002) and second waves (2006–2007) of the Young Lives Study (YLS) in Ethiopia, India (Andhra Pradesh), Peru and Vietnam, Upadhyay et al.^25^ examined the association between the use of solid cookfuels and child growth among children aged 5–76 mo. This analysis demonstrated a significant reduction in the average HAZ between the two waves in all countries except Ethiopia. However, the YLS sampling populations were not representative, and their measures of association should be interpreted with caution. None of these studies comprehensively examined how the association between solid cookfuel use and stunting in young children might vary. This is important because the mix of solid cookfuels varies by household socioeconomic characteristics and location,^20^ and maternal tobacco smoking or poor breastfeeding practices may also influence young children's growth.^26^

Our analyses showed consistently that solid cookfuel use increased the likelihood of childhood stunting in LMICs. However, the subgroup analyses suggested the magnitude of association varied according to subgroups. For example, we found that children living in households belonging to the lowest wealth quintile or households in rural areas using solid cookfuels were more likely to be stunted. Poorer households, or those in rural areas, are known to heavily rely on solid fuels for cooking.^27^ This is associated with a variety of reasons, including the cost of transitioning from solid cookfuels to modern, safe and efficient cookfuels (i.e. moving up the ‘energy ladder’).^28^ Our analysis also found that children who lived in households using solid cookfuels and had mothers who smoked tobacco were more likely to be stunted. Second-hand tobacco smoke is known to contain harmful pollutants that are reported to delay skeletal development.^29^ Therefore, solid cookfuel use and tobacco smoke combined could lead to a greater likelihood of child stunting. Stratified estimates of the association by birth weight were very similar and not viewed to be clinically significant.

Solid cookfuel use may impede children's growth through several mechanisms that reflect either a direct effect of airborne particulate exposure on growth or indirectly through increased morbidity. Household solid-fuel combustion produces relatively high levels of polycyclic aromatic hydrocarbons, which have been recognised as endocrine-disrupting chemicals, compromising endocrine system processes involving growth hormone and insulin-like growth factors.^11^ Children living in households using solid cookfuels are also more likely to develop acute respiratory infections.^9^ Repeated episodes of respiratory infections can impair growth, through increased metabolic requirements, anorexia and reduced dietary intake, increased catabolism and deranged metabolism of key nutrients.^8^

This analysis has several limitations, which should be considered when interpreting our findings. First, DHS are cross-sectional and therefore it was not possible to establish a temporal relationship between solid cookfuel use and child stunting. Second, while reporting solid cookfuels is a good proxy for exposure to smoke from cooking,^30^ future studies should be designed to conduct complex exposure assessments. Better quantification of exposure to solid cookfuel combustion products will be necessary for an improved understanding of the exposure–response relationships. Third, there is also the possibility of exposure misclassification from fuel stacking, or household air pollution to outdoor air pollution through cross-ventilation. However, misclassification from fuel stacking would result in an underestimation of the association of interest. Additionally, use of solid cookfuel stoves in the household are typically for hours and result in higher exposure to household air pollution than from outdoor sources.^31^ Fourth, several of the subgroup analyses should be viewed with caution due to missing confounder data. Finally, although our analysis controlled for several confounders, there is always potential for residual confounding. A few of the variables (such as birth weight and maternal smoking) included in the subgroup analyses may be subject to measurement error, reducing the statistical power of these analyses. In particular, the measurement of birth weight in the DHS is either from health card records or mother's recall. Due to an increased likelihood of non-facility births in LMICs, most birth weight data are from mother's recall. There is a risk of misreporting from mothers due to heaping or an inability to recall the exact birth weight. However, in the absence of more complete and accurate data, the variable included in this analysis is the most appropriate measure of birth weight. The DHS do not have appropriate measures for dietary intake and disease. We were therefore unable to examine whether these modify the association between solid cookfuel use and child stunting.

Despite these limitations, our analysis has several strengths. First, our estimates for the associations between solid cookfuel use and child stunting are based on a large and diverse sample of children from 59 nationally representative household surveys that followed standardised procedures for reporting the use of solid cookfuels. Second, we had unprecedented power and precision to examine solid cookfuel use and child stunting in LMICs and additionally examine how the association varies by subgroups.

In 2012, the WHA adopted WHO Resolution 65/6 on the Comprehensive Implementation Plan on Maternal, Infant and Young Child Nutrition, calling for combined actions in nutrition, water, sanitation and hygiene conditions to reduce childhood stunting.^32^ So far, stunting has been largely intractable to targeted interventions on complementary feeding, elimination of all diarrhoea in the first 2 y of life and water, sanitation and hygiene (WASH).^33,34^ This indicates not only the complexity of stunting but a need for a wider approach to the causes and interventions to substantially reduce the burden of childhood stunting. The WHO’s recent report (Air Pollution and Child Health: Prescribing Clean Air) highlights that the majority of children aged <5 y are exposed to household air pollution in LMICs,^35^ however, solid cookfuel use has been largely ignored by the global health community as a potentially important cause of stunting.^8^ Determining the causal role of solid cookfuel use on childhood stunting with randomised controlled trials will ultimately be needed to inform the national action frameworks to address the burden of childhood stunting.

## Conclusion

Solid cookfuel use is associated with childhood stunting in LMICs. There is an urgent need to make policymakers in the health sector, health professionals and communities aware of the deleterious association between solid cookfuel use and child stunting. To strengthen the available evidence, it is crucial that the evaluation of cookfuel intervention studies is extended to include child stunting.

## Supplementary Material

ihab090_Supplemental_File

## Data Availability

The data underlying this study are available on the DHS programme website at https://www.dhsprogram.com/.
