# Supplementary material for: Exposure to household air pollution from solid cookfuels and childhood stunting: a population-based, cross-sectional study of half a million children in low- and middle-income countries
Source: Int Health. 2022 Jan 12;14(6):639–47. doi: 10.1093/inthealth/ihab090 (PMC9623485; doi:10.1093/inthealth/ihab090)
Supplement: ihab090_Supplemental_File [file ihab090_supplemental_file.docx]

**S1 Table**. Primary cookfuels used in LMICs

| Cookfuel type | N | (%) |
| --- | --- | --- |
|  |  |  |
| Electricity | 19,695 | 3.5 |
| Liquefied petroleum gas (LPG) | 106,953 | 19.2 |
| Natural gas | 8,744 | 1.6 |
| Biogas | 10,447 | 1.9 |
| Kerosene | 10,081 | 1.8 |
| Coal lignite | 8,252 | 1.5 |
| Charcoal | 45,113 | 8.1 |
| Wood | 306,544 | 55.0 |
| Straw shrubs | 8,272 | 1.5 |
| Agricultural crop | 8,745 | 1.6 |
| Animal dung | 24,100 | 4.3 |
| Cardboard/paper/sawdust/woodchips | 150 | 0.0 |
| Other electrical source | 2 | 0.0 |

**S2 Table**. Proportion of children exposed to household air pollution from cookfuels by country

| Country | Solid cookfuel use (N, %) |
| --- | --- |
|  |  |
| Albania | 952 (38.7) |
| Armenia | 46 (2.9) |
| Angola | 3770 (59.9) |
| Azerbaijan | 300 (15.3) |
| Bangladesh | 5529 (86.2) |
| Burkina Faso | 6479 (98.4) |
| Benin | 110.94 (96.7) |
| Bolivia | 3071 (40.0) |
| Burundi | 6010 (99.8) |
| DR Congo | 7991 (98.9) |
| Congo | 3840 (89.9) |
| Cote D'Ivoire | 2942 (93.4) |
| Cameroon | 3517 (82.7) |
| Colombia | 46 (0.3) |
| Dominican Republic | 481 (15.7) |
| Ethiopia | 8298 (94.7) |
| Gabon | 1164 (34.8) |
| Ghana | 2299 (86.1) |
| Gambia | 3145 (99.7) |
| Guinea | 3363 (98.8) |
| Guatemala | 8712 (75.1) |
| Guyana | 388 (24.0) |
| Honduras | 6805 (70.5) |
| Haiti | 5405 (97.7) |
| India | 153207 (69.7) |
| Jordan | 0 (0.0) |
| Kenya | 17204 (73.5) |
| Cambodia | 3514 (81.1) |
| Comoros | 2090 (82.7) |
| Kyrgyz Republic | 1364 (35.3) |
| Liberia | 3163 (100.0) |
| Lesotho | 901 (72.2) |
| Morocco | 4659 (85.9) |
| Moldova | 151 (11.7) |
| Madagascar | 5184 (99.7) |
| Mali | 8151 (99.0) |
| Maldives | 22 (0.9) |
| Malawi | 5059 (98.9) |
| Mozambique | 9063 (97.1) |
| Nicaragua | 4608 (77.6) |
| Nigeria | 8917 (79.9) |
| Niger | 4824 (98.5) |
| Namibia | 1263 (70.7) |
| Nepal | 1701 (78.0) |
| Peru | 3954 (44.4) |
| Pakistan | 2298 (57.2) |
| Rwanda | 3527 (99.9) |
| Sierra Leone | 4297 (99.9) |
| Senegal | 5211 (89.0) |
| Sao Tome and Principe | 1319 (83.2) |
| Eswatini | 1518 (75.5) |
| Chad | 9649 (97.5) |
| Togo | 3030 (96.4) |
| Tajikistan | 944 (16.5) |
| Timor-Leste | 5112 (87.4) |
| Tanzania | 8462 (98.2) |
| Uganda | 4293 (99.7) |
| Yemen | 5177 (38.1) |
| South Africa | 310 (28.7) |
| Zambia | 8108 (94.6) |
| Zimbabwe | 3285 (68.8) |
